# Diet in the Management of Weight Loss

**DOI:** 10.3390/nu13041306

**Published:** 2021-04-15

**Authors:** Vicente Martínez-Vizcaíno, Celia Álvarez-Bueno, Iván Cavero-Redondo

**Affiliations:** 1 Health and Social Research Center, Universidad de Castilla-La Mancha, 16071 Cuenca, Spain; Celia.AlvarezBueno@uclm.es (C.Á.-B.); ivan.cavero@uclm.es (I.C.-R.); 2 Facultad de Ciencias de la Salud, Universidad Autónoma de Chile, Talca 3460000, Chile; 3 Universidad Politécnica y Artística del Paraguay, Asunción 001518, Paraguay; 4 Rehabilitation in Health Research Center (CIRES), Universidad de las Americas, Av. Republica 71, Santiago 72819, Chile

The prevalence of obesity and related disorders has been growing at an alarming rate in both wealthy and middle–low-income countries. Therefore, epidemics of communicable diseases aside, obesity remains an urgent public health priority that has its roots in an energy imbalance, and that can only be tackled from two complementary perspectives: increasing energy expenditure through physical activity and modifying energy intake.

Numerous diet-based initiatives across the world have been implemented for the management of weight loss, but very few of them have shown some effectiveness in the medium–long term. This Special Issue included articles addressing a variety of approaches to improve the understanding of the difficulties to manage the weight loss, as well as some innovative interventions to achieve the weight in a sustained way. Thus, two studies involve qualitative methods to identify barriers to the adherence to weight loss diets [1,2], two provide analyses of randomized controlled trials (RCT) [3,4] and four are systematic reviews [5,6,7,8].

The qualitative study of De Leon, A. [1] et al. represents a valuable and innovative contribution to the understanding of the barriers to adherence to weight loss diets for two reasons: they are exclusively focused in women and used nominal group techniques as the strategy to identify and prioritize the perceived barriers to adherence to a weight loss diet. Their conclusions highlight that both individual and environmental factors are involved in a woman’s decision to abandon a diet; among them, the lack of information of acceptable portion sizes, as well as difficulties to deal with emotional and stress eating were noted as important issues.

With a mixed design involving both quantitative and qualitative methods, Cruwys, T. et al. [2] examined the factors that predict the adherence to five restriction based dietary patterns (weight loss, vegan, vegetarian, paleo and gluten free). They reported that the adherence of gluten-free and weight-loss dieters was poorer than those following vegan and vegetarian diets. Social identification and self-efficacy appear as factors that positively influenced the adherence to these diets.

The RCT of Röhling, M. et al. [4] evaluates the effectiveness of a multi-component, occupational healthcare program SHAPE-AND-MOTION-Medical-Accompanied-Slimming (SAMMAS) whose objective aims to lower daily insulin levels as a way to increase lipolysis. This strategy seems to represent a promising approach to reduce body weight and to maintain this reduction in the long term. Other study analyzing the PREDIMED-Plus randomized trial [3] assesses whether the changes in diet quality depends on the reported preceding maximum weight; this study concluded that those participants who enter the PREDIMED-Plus at their maximum weight were those in which the intervention is most efficacious in the short term.

The four systematic reviews included in this Special Issue provided evidence on the effectiveness of several interventions. First, the study of Alsharif, F.J. et al. [5] reviews the beneficial effects of curcumin supplementation on reducing weight in overweight or obese adults through the regulation of lipid metabolism and the limitation of the effect of pro-inflammatory cytokines. The systematic review of Cavero-Redondo et al. [6] provides evidence supporting that the mHealth self-monitoring of diet and physical activity behaviors are appropriate interventions for weight management in adults with excess of weight because the adherence to this type of interventions is greater than for other ones. The study of Godoy-Cumillaf, A. et al. [7], meanwhile, compares the effect on BMI of physical activity only interventions with those physical activity plus diet interventions in children and adolescents, concluding that physical activity plus diet interventions are able to reduce BMI, but physical activity only interventions did not. Finally, Lakicevic, N. et al. [8] et al., reviewing the evidence on the impact of the rapid weight loss in judo athletes, warn about the importance of monitoring the minimal competitive weight of athletes prioritizing the health over the benefits in the sport.

Taken together, this Special Issue provides a comprehensive overview of dietary management in weight loss because it includes the most diverse perspectives on how to approach this common clinical consultation issue for endocrinologists, nutritionists and primary care physicians and nurses.

## Data Availability

Not applicable.

## References

[1] De Leon A., Roemmich J.N., Casperson S.L. (2020). Identification of Barriers to Adherence to a Weight Loss Diet in Women Using the Nominal Group Technique. Nutrients.

[2] Cruwys T., Norwood R., Chachay V.S., Ntontis E., Sheffield J. (2020). “An Important Part of Who I am” The Predictors of Dietary Adherence among Weight-Loss, Vegetarian, Vegan, Paleo, and Gluten-Free Dietary Groups. Nutrients.

[3] Bouzas C., Bibiloni M.D.M., Garcia S., Mateos D., Martínez-González M.Á., Salas-Salvadó J., Corella D., Schröder H., Martínez J.A., Alonso-Gómez Á.M. (2020). Dietary Quality Changes According to the Preceding Maximum Weight: A Longitudinal Analysis in the PREDIMED-Plus Randomized Trial. Nutrients.

[4] Röhling M., Martin K., Ellinger S., Schreiber M., Martin S., Kempf K. (2020). Weight Reduction by the Low-Insulin-Method—A Randomized Controlled Trial. Nutrients.

[5] Alsharif F., Almuhtadi Y. (2021). The Effect of Curcumin Supplementation on Anthropometric Measures among Overweight or Obese Adults. Nutrients.

[6] Cavero-Redondo I., Martinez-Vizcaino V., Fernandez-Rodriguez R., Saz-Lara A., Pascual-Morena C., Álvarez-Bueno C. (2020). Effect of Behavioral Weight Management Interventions Using Lifestyle mHealth Self-Monitoring on Weight Loss: A Systematic Review and Meta-Analysis. Nutrients.

[7] Godoy-Cumillaf A., Fuentes-Merino P., Díaz-González A., Jiménez-Díaz J., Martínez-Vizcaíno V., Álvarez-Bueno C., Cavero-Redondo I. (2020). The Effects of Physical Activity and Diet Interventions on Body Mass Index in Latin American Children and Adolescents: A Systematic Review and Meta-Analysis. Nutrients.

[8] Lakicevic N., Roklicer R., Bianco A., Mani D., Paoli A., Trivic T., Ostojic S.M., Milovancev A., Maksimovic N., Drid P. (2020). Effects of Rapid Weight Loss on Judo Athletes: A Systematic Review. Nutrients.

